# Low coverage sequencing of two Asian elephant (*Elephas maximus*) genomes

**DOI:** 10.1186/2047-217X-3-12

**Published:** 2014-07-10

**Authors:** Akbar Dastjerdi, Christelle Robert, Mick Watson

**Affiliations:** 1 Virology Department, Animal Health and Veterinary Laboratories Agency, Weybridge, KT15 3NB Addlestone, Surrey, UK; 2 The Roslin Institute and Royal (Dick) School of Veterinary Studies, University of Edinburgh, EH25 9RG Easter Bush, UK; 3 Edinburgh Genomics, University of Edinburgh, EH25 9RG Easter Bush, UK

**Keywords:** Elephant, Genome, Sequencing, Variation

## Abstract

**Background:**

There are three species of elephant that exist, the Asian elephant (*Elephas maximus*) and two species of African elephant *(Loxodonta africana* and *Loxodonta cyclotis)*. The populations of all three species are dwindling, and are under threat due to factors, such as habitat destruction and ivory hunting. The species differ in many respects, including in their morphology and response to disease. The availability of elephant genome sequence data from all three elephant species will complement studies of behaviour, genetic diversity, evolution and disease resistance.

**Findings:**

We present low-coverage Illumina sequence data from two Asian elephants, representing approximately 5X and 2.5X coverage respectively. Both raw and aligned data are available, using the African elephant (*L. africana)* genome as a reference.

**Conclusions:**

The data presented here are an important addition to the available genetic and genomic information on Asian and African elephants.

## Data description

### Background

Three species of elephant exist, the Asian elephant (*Elephas maximus*) [1] and two species of African elephant *(Loxodonta africana*, *Loxodonta cyclotis)*[2,3]. Elephants are the Earth's largest land mammal, with the Asian elephant (*E. maximus*) being slightly smaller than African elephants (*L. africana* and *L. cyclotis*). Elephants can reach a height of 4 m, a weight of over 10,000 kg, and adults can have a lifespan as long as 60–70 years. Asian elephants have the following distinctive characteristics: smaller rounded ears, arched back, hemispherical bulges on the head, differing number of nails on the legs, and finger-like features on the trunk. Asian elephant males and both sexes of African elephants can grow tusks over 2 m in length and 45 kg in weight. Asian elephants also have fewer ribs, more molar plates and a small intestine twice as long as that in African elephants [1,2,4]. The elephant species also differ in their nutritional needs [5] and susceptibility to infectious diseases [6].

Characteristic features of elephants make these animals unique. Elephant trunks have many functions (smelling, breathing, trumpeting, drinking and grabbing) and contain approximately 150,000 different muscle fascicles [7], the largest number in any single body part. Elephants have also a longer gestation period than any other mammal, almost 22 months [8]. At birth, elephant calves already weigh some 90 kg and stand about 1 m tall.

Both *E. maximus* and *L. africana* are listed as endangered species by the International Union for Conservation of Nature (IUCN) [9]. Wild populations are restricted to increasingly limited areas of land and are under pressure with regard to habitat loss, poaching, human invasion and various diseases.

The genome of the African elephant, *L. africana*, has been sequenced to 6.8X coverage by The Broad Institute [10] (Genbank Assembly ID: GCA_000001905.1; BioProject accession ID: PRJNA12569) and has been annotated by Ensembl [11]. Here, we present whole-genome sequencing datasets from two Asian elephants (*E. maximus*), both of which are an important addition to the existing public data. The availability and a better understanding of elephant genomes will facilitate efforts to conserve populations of these animals by enabling studies of behaviour, social organisation, population structure, genetic diversity and phylogeny. In addition, the genome sequences will facilitate development of novel tools to aid in combating the illegal trade of wild elephant and ivory, as well as deciphering their body physiology and immunity.

### Original purpose

Two samples, 577_1_Emelia and 577_2_Raman, were originally sequenced to enable the characterisation of elephant endotheliotropic herpesvirus (EEHV) [12], which is associated with a life-threatening haemorrhagic disease in Asian elephants [6]. Despite the virus representing only a tiny fraction of the reads (0.169% and 0.038% respectively), full genome sequences of both EEHV1A and EEHV1B were recovered. A small number of the reads were also used in the resolution of the type specimen of the Asian elephant (*E. maximus*), which was instead found to originate from an elephant of the *Loxodonta* genus [13].

### Sample treatment and sequencing

Sample collection, treatment and sequencing have been previously described [12]. Briefly, post-mortem samples were collected from two infant Asian elephants, Emelia (female) [14] and Raman (male) [15], from two UK zoos. The individuals were a similar age at death: 2 years 8 months 11 days (Raman) and 2 years 9 months (Emelia). DNA was extracted from heart (Raman) and tongue (Emelia). From each sample, 5 μg of DNA was treated with NEBNext double-stranded DNA fragmentase (New England BioLabs, Ipswich, MA). Sequencing libraries were constructed using an Illumina TruSeq DNA sample preparation kit. The libraries were sequenced on an Illumina HiSeq 2000 (Illumina, San Diego, CA) instrument at Edinburgh Genomics (University of Edinburgh), resulting in 76 bp paired-end datasets.

### Bioinformatics, data description and availability

The data are deposited in the European Nucleotide Archive (ENA) under accession [EMBL:ERP004241], and consist of 97.6 million (577_1_Emelia) and 53.8 million (577_2_Raman) paired 76 bp reads. Assuming a genome size of 3.1Gb, the sequence data described here represent 4.8X and 2.6X theoretical coverage respectively. The insert size for each library is approximately 350 bp. The reads have been aligned to the African elephant genome (Loxafr3.0, INSDC Assembly GCA_000001905.1, Jul 2009) using bwa [16] and converted to BAM by Samtools [17]. The vast majority of the Asian elephant sequence reads align to the African elephant genome: 94.66% (577_1_Emelia) and 93.82% (577_2_Raman). The percentage of bases in the *L. africana* assembly covered by at least one read from the Asian samples are 94.18% and 84.76% for 577_1_Emelia, and 577_2_Raman respectively. These coverage percentages were calculated using the mpileup tool from Samtools [17] package (version 0.1.18) with the option –C set to 50.

## Availability of supporting data

The datasets supporting the results of this article are available in the European Nucleotide Archive (ENA) under accession [EMBL:ERP004241], as well as the *GigaScience*, GigaDB repository [18].

## Abbreviations

Bp: Base-pairs; EEHV: Elephant endotheliotropic herpesvirus; Gb: Gigabases; IUCN: International Union for Conservation of Nature; kg: Kilograms; m: Metres.

## Competing interests

The authors declare they have no competing interests.

## Authors’ contributions

AD conceived the study, provided samples and helped write the paper; CR carried out bioinformatics analysis and helped write the paper; MW conceived the study, carried out bioinformatics analysis and helped write the paper. All authors read and approved the final manuscript.
